# The Role of Positive 3-H Words in Peacebuilding and Engagement in EFL/ESL Classes

**DOI:** 10.3389/fpsyg.2021.745645

**Published:** 2021-08-27

**Authors:** Xiaojing Shi

**Affiliations:** Center for Second Language Writing Research/School of College English Teaching and Research, Henan University, Kaifeng, China

**Keywords:** positive 3H, peacebuilding, engagement, emotions, English as a second/foreign language

## Abstract

This review aimed at investigating the role of help, hope, and harmony formally known as positive 3H on students' engagement and peacebuilding. This topic has recently attracted attention since teachers and the way they treat students are said to play a paramount role in the learning process and as a result, peace can be built in the classroom and students also are more likely to be actively engaged in the tasks. To start with, a definition for positive 3-H was presented coupled with the role it plays in language learning contexts. Then the way both inner peace and interpersonal peace have been applied in the learning process to build peace is discussed. Following that, the effects of positive 3-H on students' engagement and peacebuilding through raising some relevant activities are dealt with. Finally, implications and further directions are put forward.

## Introduction

People's hearts and minds are found to be undoubtedly affected by language and words, and the effect might be either short-term or long-term (Lewandowska-Tomaszczyk et al., 2020). It has always been people's natural desire to feel connected to a community and fit in with a group of people; therefore, long-lasting relationships can be strongly dependent on the amount to which a person can communicate interpersonally (Holmes, 2008). In terms of positive 3-H and peacebuilding, a cognitive dimension of learning is involved as well as a linguistic one. Positive 3-H (Help, Hope, and Harmony) (Curtis and Oxford, 2021) indicates how individuals can thrive and be happier, concentrating on positive emotions like hope, enthusiasm, resilience, positivity, and so forth rather than negative feelings (Dewaele, 2015; MacIntyre et al., 2019; Li, 2020; Li et al., 2020). The process of deeper learning is affected by learners' variables such as well-being, self-efficacy, etc. Moreover, students reach the expected consequences of education as long as they are provided with a positive ambiance coupled with a rapport between teachers and students throughout the class that can be regarded as a social context since it is a microcosm of society. Having given that a language learning situation where students have to struggle with learning a new language and practices through which peace can be built seem crucially significant. These mentioned practices have been conceptualized in both positive psychology and positive peace psychology characterized by the way people thrive and the amount they can actively build peace rather than growing negative senses within themselves (Gibson, 2011; MacIntyre and Mercer, 2014). The present article aims to scrutinize three positive aspects of classroom language (i.e., help, hope, harmony) and their effects on both peacebuilding and students' engagement in the classroom context. For more clarification, some activities, through which peace can be built and negative emotions can be reduced, have been added as well.

## Background

### Definitions of the Positive 3-H Words

Education is not merely giving students information; instead, through the given information, students' lives should be harmonized. The phrase Positive 3H (Curtis and Oxford, 2021) refers to hope, help, and harmony. Regarding the pivotal role of positive emotions, many studies have proven the fact that a feeling of being engaged, being motivated, being successful, being efficacious, being interested, accomplishing one's goals, performance, and so forth, are enhanced (Derakhshan et al., 2019; MacIntyre et al., 2019; Li, 2020). To explicate it more, “Hope” has been defined in positive psychology in a cognitive theory as (**a**) an individual's belief in his capabilities and as a consequence of it, mental goals and desired future aims can be envisioned, paving the way to move toward one's goals and (**b**) the ability and motivation to start and feel determined as they are striving hard to achieve their goals. According to Snyder's model, the higher the levels of enduring hope, the greater the academic entrance exam scores and the higher the self-esteem would be. As a matter of fact, the process of learning a new language is anxiety-provoking, and it is the teachers' hopeful comments that help students not to lose hope (Snyder, 2000). In terms of “Help,” the considerable role of teachers is emphasized because through their caring help and encouraging words, students' anxiety can be assuaged and weakened; accordingly, students' communicative abilities can be expressed to the people by whom they are surrounded. It can vividly be interpreted that a rapid decline in students' motivation can be halted as challenging situations along with supportive, helpful encouragement are provided by both the teachers and mentors (Curtis and Oxford, 2021). “Harmony” refers to people living and working together without fighting and disagreeing with each other. Empathy, which means the experience of people's feelings and thoughts, has been shown to be the stepping stone to human “Harmony” (Oxford, 2016). Another aspect from which harmony can be taken into consideration is change since learning a new language needs one to give up his comfort zone. Change that is extremely stressful at times can be experienced by the students who are seriously studious since change challenges the items which are familiarized to us and thus, one's normal needs for safety, being comfortable, and having control over what has been done are questioned. Despite the fact that no one likes to experience the feeling of being disturbed within themselves, it is an inseparable part of growing (Singer, 2007).

### The Definition and Application of Negative 3-H Words

As for positive emotion, Curtis and Oxford (2021) raised another taxonomy in which hateful, hurtful, and harmful language is stressed. They might seem synonymous at the first glance; however, they can be used in their own contexts. “Hate” refers to a feeling of dislike. It can be shown in many communicative contexts from personal to societal. A serious argument, for example, is caused by using a hateful language. “Hurt” and “Harm” seems the same in that both bring about physical and emotional injury, yet the point is that the degree of injury would be higher when harming someone. Similarly, hurt sometimes has a positive effect, while harm does not.

One of the activities that can be put into practice is using insults in L1 so as to stop using them in L2. Care should be taken so as not to offend anyone in the class since the words are so insulting that they cannot be used in public. According to Dewaele (2011) teachers perceive that both boredom and anxiety are the most important sources for lack of improvement in foreign language learning that may be caused by being hurt in the learning context. It is hence not surprising that researchers in SLA and in language teaching research have concentrated on ways to provide a positive and stimulating learning environment in order to engage learners and enhance their language learning motivation (Dewaele, 2011).

### Applying Peacebuilding for Inner Peace

When learning a new language, the students' aim is two-fold: to acquire knowledge about the language and speak it fluently and to raise educational knowledge to teach foreign language learners; thus, the learning process is impacted by the emotions either positively or negatively (Swain, 2011). Students' motivations, attention, and self-regulation either favorably or unfavorably are the emotions that are affected through this process. In recent years, emotions have received a more significant role in academic studying as they have been demonstrated to affect students' persistence and the strategies applied for it, students' engagement, learning, and accomplishments. So considering positive 3-Hs and positive psychology in which improving positive emotions, increasing engagements, appreciating meaning in life are highlighted, positive emotion makes learning easier since it has a tendency to widen one's horizon, therefore resulting in an increase in attention, creativity, and thriving (Fredrickson, 2001).

Furthermore, students' emotions can be managed and regulated so that positivity would be ameliorated. Regulating emotion can be enhanced through a conversation with people who are more capable either peers or teachers (Vygotsky, 1978). With regard to this method, a goal is activated, strategies are opted for and applied, an outcome is identified, and consequently, emotions can be regulated. Regarding that, Inner peace is linked to a person's emotions, it can be easily jeopardized by internal stressors, such as the lack of emotion regulation and self-direction, in addition to external factors (Oxford, 2017a,b). It can be taken as an example, university students put a strain on themselves to achieve specific goals and they are expected not to fail (Pekrun et al., 2002). These are the internal threats that may lead to unpleasant learning results. With regard to the above example, negative emotions cannot often be managed successfully and may put both inner peace and well-being in danger.

The two main categories of peace in L2 education are inner peace and interpersonal peace; the former is concerned with one's peace within himself, and the latter is peace with whom you know. Inner peace was defined as a state of well-being that does not depend on the presence of external or internal pleasurable stimuli. In the L2 learning context, inner peace is relevant to learners' characteristics and self-concept which is about the beliefs such as self-esteem, self-confidence, and self-efficacy that learners have about their abilities and how they evaluate their competence in the context of L2 learning (Williams et al., 2016). Self-concept can also be influenced by friendly teachers supporting their students so that the students can be encouraged to learn from their mistakes so both self-esteem and self-confidence can be boosted in the students in this way; hence, they develop their beliefs about themselves even faster contributing to enhancing their inner peace.

### The Effect of Positive 3-H on Students' Engagement and Peacebuilding

Engagement refers to the amount learners participate actively in the activities. Engaged learners can be responsible for their learning and it is unlikely to attain meaningful learning without being engaged. Engagement is said to be context-specific, for instance, learners' culture, family, school, peers affect their engagement (Hiver et al., 2021). Learner engagement is a complex construct consisting of multiple dimensions which together contribute to successful learning. Wang and Derakhshan (2021) highlighted that affective, social, behavioral, and cognitive dimensions of engagement are interrelated, leading to the improvement of particular habits and attitudes toward learning. Most language professionals have reached the agreement that for communicative skills to be mastered, participation is the key to success and it takes learners' passionate engagement (MacIntyre and Gregersen, 2012). What can be pinpointed is that disengagement gives rise to a reduction in positive emotions, such as self-esteem and self-regulation and an increase in helplessness (Derakhshan, 2021).

Some activities have been created to involve the students and help them build peace. According to Gregersen and MacIntyre (2021), the activities should not be necessarily content-specific, but a positive psychological context should be set that could cause more meaningful learning. One of the organized activities is classified into three phases: forethought, performance, reflection. In the first phase, the task was analyzed by the students to understand and be alert about the emotions that were felt, throughout oral production. During the second phase, they were persuaded to monitor their performance and the focus was on the experienced emotions during the task; as a result, they could perceive how to regulate the emotions. Through the last phase, they were promoted to estimate how these strategies aid them in finding inner peace (Oxford, 2017b).

### Implications and Future Directions

As emotions are of great importance in building one's character and helping one develop a sense of tranquility to learn a new language, this study has focused on positive emotions known as positive 3-H words which facilitate the process of learning. Consequently, there are a few restrictions that should be kept in mind and should also be addressed in future studies. Even though emotions are said to play a paramount role in the process of learning, surprisingly it has received little attention. Had students been provided with helpful ideas, a harmonious learning situation, and hopeful comments and encouragement, peace could have been built through the process of learning and anxiety would have been lowered. Studies have been conducted to collect information about what types of emotions impede the process of learning; however, a few studies were carried out to describe a learning atmosphere in which positive 3-H words are applied in all aspects, and building peace is the most crucial priority. Honestly, when peace is built, most of the negative feelings, impeding the learning process, will be diminished and thus, the process of learning will be facilitated. As it has been explained, both linguistic and cognitive dimensions should be taken into account if we expect one to grow faster in terms of personality and grasping a new language. So from an institutional point of view, teachers should be trained to utilize positive 3-H words while teaching so that students feel blissfully happy and they can cope with stress which arises while learning a new language. Yet, high engagement is highly unlikely to be achieved through classes. Nonetheless, as it was proposed by Schlechty (2011), one which is not hard to achieve is that most students should be involved most of the time. In this regard, researchers should conduct both qualitative and longitudinal studies for monitoring these activities applied through the class and their long-term effects on students' educational and personal lives. Additionally, thanks to state-of-the-art technology and a fast-changing world, a new way of interacting between teachers and students has been put forward, online classes, so emphasis should be laid on how and why anxiety is experienced during such classes.

## Author Contributions

The author confirms being the sole contributor of this work and has approved it for publication.

## Conflict of Interest

The author declares that the research was conducted in the absence of any commercial or financial relationships that could be construed as a potential conflict of interest.

## Publisher's Note

All claims expressed in this article are solely those of the authors and do not necessarily represent those of their affiliated organizations, or those of the publisher, the editors and the reviewers. Any product that may be evaluated in this article, or claim that may be made by its manufacturer, is not guaranteed or endorsed by the publisher.
